# Bronchiectasis Information and Education: a randomised, controlled feasibility trial

**DOI:** 10.1186/s13063-020-4134-5

**Published:** 2020-04-15

**Authors:** Katy L. M. Hester, Vicky Ryan, Julia Newton, Tim Rapley, Anthony De Soyza

**Affiliations:** 1 Population Health Sciences Institute, Newcastle University, Newcastle Upon Tyne, NE1 7RU UK; 2 Adult Bronchiectasis Service, Freeman Hospital, Newcastle upon Tyne Hospitals, Newcastle upon Tyne, NE7 7DN UK; 3 Faculty of Medical Sciences, Newcastle University, Newcastle upon Tyne, NE2 4HH UK; 4 Department of Social Work, Education and Community Wellbeing, Northumbria University, Newcastle upon Tyne, NE7 7XA UK

**Keywords:** Bronchiectasis, Exacerbation, Self-management, Education, Information, Randomised controlled trial, Feasibility study, Qualitative research

## Abstract

**Background:**

There has been comparatively little patient information about bronchiectasis, a chronic lung disease with rising prevalence. Patients want more information, which could improve their understanding and self-management. A novel information resource meeting identified needs has been co-developed in prior work. We sought to establish the feasibility of conducting a multi-centre randomised controlled trial to determine effect of the information resource on understanding, self-management and health outcomes.

**Methods/design:**

We conducted an unblinded, single-centre, randomised controlled feasibility trial with two parallel groups (1:1 ratio), comparing a novel patient information resource with usual care in adults with bronchiectasis. Integrated qualitative methods allowed further evaluation of the intervention and trial process. The setting was two teaching hospitals in North East England. Participants randomised to the intervention group received the information resource (website and booklet) and instructions on its use. Feasibility outcome measures included willingness to enter the trial, in addition to recruitment and retention rates. Secondary outcome measures (resource use and satisfaction, quality of life, unscheduled healthcare presentations, exacerbation frequency, bronchiectasis knowledge and lung function) were recorded at baseline, 2 weeks and 12 weeks.

**Results:**

Sixty-two participants were randomised (control group = 30; intervention group = 32). Thirty-eight (61%) were female, and the participants’ median age was 65 years (range 15–81). Median forced expiratory volume in 1 s percent predicted was 68% (range 10–120). Sixty-two of 124 (50%; 95% CI, 41–59%) of potentially eligible participants approached were recruited. Sixty (97%) of 62 participants completed the study (control group, 29 of 30 [97%]; 95% CI, 83–99%; 1 unrelated death; intervention group, 31 [97%] of 32; 95% CI, 84–99%; 1 withdrawal). In the intervention group, 27 (84%) of 32 reported using the information provided, and 25 (93%) of 27 of users found it useful, particularly the video content. Qualitative data analysis revealed acceptability of the trial and intervention. Web analytics recorded over 20,000 page views during the 16-month study period.

**Conclusion:**

The successful recruitment process, high retention rate and study form completion rates indicate that it appears feasible to conduct a full trial based on this study design. Worldwide demand for online access to the information resource was high.

**Trial registration:**

ISRCTN Registry, ISRCTN84229105. Registered on 25 July 2014.

## Background

Bronchiectasis is a chronic lung condition of increasing worldwide prevalence [1–3]. In the United Kingdom, for example, prevalence is between 43.4/100,000 in those aged 18–30 and 1239.7/100,000 in those aged 70–79 [1]. In addition, there is evidence that up to 50% of patients with chronic obstructive pulmonary disease (COPD) have co-existent bronchiectasis [4, 5], and as such, case finding of bronchiectasis is likely to rise further. This rising prevalence presents a large burden to healthcare service providers [6, 7]. The nature of bronchiectasis, with dilated bronchi leading to symptoms of breathlessness, chronic productive cough and intermittent infective exacerbations, presents a significant burden to patients and their families. Patients often have recurrent, costly hospital admissions; a poorer quality of life [8, 9]; and clinically significant fatigue [10, 11].

Current treatments for bronchiectasis include oral, inhaled or intravenous antibiotics; mucolytics; and physiotherapy [12]. Timely recognition of symptoms and improved management of infections could lead to increased disease stability in bronchiectasis and consequent improvements in health outcomes. Responding appropriately to symptoms of exacerbation and maintenance treatments such as chest clearance techniques may prevent deterioration and reduce admissions. Understanding the importance of complex treatment regimens can be challenging for patients; yet, poor adherence to treatments, such as inhaled antibiotics, has been shown to worsen outcomes and exacerbation rates [13, 14]. It follows, therefore, that patient self-care could make a significant impact on healthcare service use. To enable patients to self-care, access to accurate information about their condition is required. Such information could empower patients to recognise changes, respond to them and understand how their self-management could potentially alter their prognosis. The British Thoracic Society (BTS) guidelines for management of bronchiectasis recommend education of patients within their management plan [12]. The BTS provides a brief self-management tool for patients with bronchiectasis that is available to download. There is relatively little information produced for patients with bronchiectasis [15].

The evidence that patients with health issues want to be able to access health information and that it can influence patient outcomes is long-standing [16–19]. Specifically, in bronchiectasis, patients report feeling more confident with managing their condition following information and education about their treatment [20], and lack of information has been highlighted as one of the major barriers to self-management [21]. In addition, patients with bronchiectasis have described a lack of trustworthy information available to them beyond that obtained in the clinic and acknowledge the role of patient information in developing the skills and confidence to live with and manage their condition [22, 23].

A prior qualitative study used in-depth interviews to identify unmet information needs and priorities for an information resource [24, 25]. Using the themes and needs identified during analysis of these interviews, a novel patient information resource has been co-developed [26]. The content and format of the resource are based on the findings of the interviews and subsequent focus groups with patients and carers to refine the intervention. A definitive, multi-centre trial using this novel resource as an intervention would aim to ascertain if the provision of such patient-focussed information and education can improve health outcomes in bronchiectasis. The rationale for this feasibility study prior to a definitive trial was to assess whether the proposed design for the trial is practicable and would allow the proposed outcomes to be measured. In addition, the feasibility study allowed the intervention to be further evaluated using both quantitative and qualitative approaches.

## Objectives

The primary objective of the BRIEF (BRonchiectasis Information and Education Feasibility) study was to determine the feasibility of conducting a definitive RCT based on this trial design and establish the need for any refinements to the design or conduct of that trial. The secondary objectives of the trial were to assess and further refine the patient information resources and collect information on patient preferences. The objectives of a potential future definitive trial would be to evaluate whether provision of a patient-focussed information and education resource can improve patient understanding, self-management and health outcomes in bronchiectasis.

## Methods and design: participants, interventions and outcomes

The BRIEF study was an unblinded, single-centre, randomised controlled feasibility trial with two parallel groups, comparing a novel patient information resource with usual care in bronchiectasis. The full study protocol has previously been published, and no changes were made to the methods after trial commencement [27]. Study duration for each participant was 12 weeks from study entry date, with study visits at baseline, week 2 and week 12. Three postal record sheets were also completed during this time.

The study took place in the Newcastle upon Tyne Hospitals NHS Foundation Trust, Newcastle upon Tyne, UK. This consists of two teaching hospital sites: the Freeman Hospital and the Royal Victoria Infirmary. Study visits all took place within the Freeman Hospital. Participants were recruited from either hospital site.

Potential participants were identified by case note review and attendance at outpatient clinics and were given or sent a letter of invitation to the study and a participant information sheet. Written informed consent was obtained from willing participants. Participants could withdraw consent at any point with no effect on usual care. At the end of the study, some participants were invited to attend a focus group about their experience. Participants invited to attend the focus group were sampled purposively. The aim was to form a group that included participants of differing backgrounds and time since diagnosis, some from the control and some from the intervention group, and those who had differing preferences in terms of format of resource used. Involvement in the focus group, however, was an optional extra, and as such, a pragmatic approach had to be taken. Participants agreeing to take part in the focus group were invited to bring along their ‘carer’ (e.g., partner or family member), who was then sent the appropriate information sheet to consider whether they would like to take part. Additional information sheets and consent forms were produced for ‘carer’ participants.

To be eligible for study inclusion, participants had to have capacity to provide written informed consent, be aged 18 years or over, have a clinical and radiological diagnosis of bronchiectasis, and be able to use the English language. Exclusion criteria included having participated in the preceding Bronchiectasis Information and Education study, cognitive impairment, and being unable to use the English language.

### Study intervention

At the baseline visit, participants randomised to the intervention received the patient information resource: an overview booklet and website (password required for website access to www.bronchiectasis.me during study period). Verbal and written access instructions were given by appropriate members of the research team. Participants had access to the intervention for the duration of the study. Participants were encouraged to use the resource and to allow their families or carers to use them also. For those who did not wish to or were unable to access the website, participation using a PDF version of the information contained within the website was offered. This enabled viewing of all information except the video clips. Those in the usual care arm did not receive any additional information but were free to obtain any information routinely acquired during their usual contacts with their healthcare team or their own information seeking. At study completion, those randomised to the intervention group were allowed continued access to the resource. Those in the control group were offered access to the resource following completion of their study period. Any uptake of the resource following study completion did not form part of data collection. Any participant could choose to leave the study at any point with no effect on usual care.

### Outcome measures

The primary outcome measures for the BRIEF study were those measuring feasibility. These included participants’ willingness to enter the trial, recruitment rate, acceptability of study design and completion of required study forms and visits as per protocol. Secondary outcome measures included the measures used to evaluate the information resource and the measures that would be used within a definitive trial to assess impact of the intervention upon health-related outcomes. For the purposes of this feasibility study, these measures contributed to the assessment of completion of study forms by participants and the resource evaluation process. Measures included the bronchiectasis knowledge questionnaire (BKQ) (Additional file 3); the Resource Satisfaction Questionnaire (RSQ) (Additional file 1); and validated questionnaires such as Quality of Life–Bronchiectasis (QOL-B), Hospital Anxiety and Depression Scale (HADS), Fatigue Impact Scale (FIS), and 5-Level EuroQol 5-dimension quality of life scale (EQ-5D-5L). The number of unscheduled presentations, exacerbation rate and forced expiratory volume in 1 s (FEV_1_) could potentially be used in a future full trial as a representation of the patients’ ability to self-manage their condition. Health-related quality of life (HRQOL) measures were also felt to be important to include in a future definitive trial and were therefore recorded in this study. A recent review of their use in bronchiectasis has shown that they have good validity and repeatability. Specifically, QOL-B and St George’s Respiratory Questionnaire were identified as having good psychometric properties, and QOL-B is the only disease-specific HRQOL questionnaire available for bronchiectasis [28]. Full details are included in the pre-published study protocol [27].

Outcome measures were recorded at baseline (day 0) and then at 2 weeks (i.e., shortly after initial viewing of information in order to facilitate obtaining first opinions) and 12 weeks after recruitment. This was done during participant visits that took less than 1 h each. Visit 2 could be conducted via telephone. Participants were additionally asked to complete three postal record sheets (weeks 4, 8 and 12), enabling identification of episodes of change in symptoms and information resource use.

In addition, at the end of the study, participants (and their carers) were invited to a focus group to discuss both the resource itself and the trial process. Whilst the trial was running, the website use was also monitored using basic analytics to determine page views, navigation through the site and attempts at access from those outside of the study.

#### Sample size

Sample size was 70, with a minimum of 30 being randomised to each arm. This was based on previous recommendations for good practice in feasibility studies [29]; no formal power calculations were carried out. Up to ten ‘carer’ participants were anticipated to be recruited for the end of study focus group, as described above.

It was estimated that 24 months would be adequate time to recruit 70 participants to this study, based on a clinic attendance of approximately 60 per month with an estimate of 50% of participants approached who were willing and able to enter. Seventy participants recruited from among approximately 140 participants approached would correspond to a 95% CI for the recruitment rate of 41–59% (an acceptable width of ± 9%). We expected low attrition rates based on previous work and our prior experience in this field. There was a 3-month additional period planned for follow-up of the last recruited participants and time beyond for the focus group and analysis.

#### Randomisation

Participants were randomised to intervention or control groups in a 1:1 ratio, using variable length random permuted blocks within strata. Because there was a female preponderance within the potential study population and the effect of gender on outcomes was uncertain, randomisation was stratified by gender. The randomisation allocation schedule was generated by a statistician with no other involvement in the study. Randomisation was performed by the Chief Investigator at site, or by an individual with delegated authority, using a secure, password-protected, web-based system administered by Newcastle Clinical Trials Unit. Blinding was not feasible for this study for participants or the research team conducting the study visits, owing to the nature of the intervention.

#### Statistical methods

Analyses were carried out according to a pre-defined statistical analysis plan (SAP). Analyses were based on the intention-to-treat (ITT) principle with analysis groups based on the groups allocated at randomisation, with all randomised participants being included in the analyses. The analyses of the data collected were descriptive, and no formal statistical testing was performed. For the feasibility objectives 95% CIs have been reported [30, 31]. Validated questionnaires were scored using the scoring algorithm provided; pre-determined rules for missing data were followed. For those questionnaires that were either unvalidated or had no pre-defined rules for dealing with missing data, missing data points were treated as such. The extent of missing data was assessed and reported, and analyses of outcomes at follow-up time points were carried out on a complete-case basis. Rates were calculated as defined. At baseline and by intervention group, the distribution of all numerical variables was examined graphically and summarised by the median and range. Baseline categorical variables were tabulated, and percentages reported. The changes from baseline to 2 weeks and baseline to 12 weeks, for all validated health-related and quality of life outcomes, have been reported as means and SDs, the purpose being to inform the design of any future definitive trials (Table 3). No inferential analyses have been performed, because the study was not powered to do so. Not all the analyses proposed in the published protocol paper [27] were included in the SAP. For the secondary outcomes, CIs for the estimated difference in the change from baseline between intervention groups have not been reported. Statistical analyses were performed using IBM SPSS Statistics version 22.0 software (IBM Corp., Armonk, NY, USA).

The focus group conducted at the end of the study explored the experiences of participants and their carers. Topics covered both their views on the resource and their views on the trial itself. A thematic analysis approach was taken [32–34]. The group discussion was audio-recorded and transcribed verbatim, and transcripts were used to read, re-read and confirm issues identified during the focus group. Feedback themes were identified, summarised and reported.

## Results

### Participant recruitment and flow

**Fig. 1:**
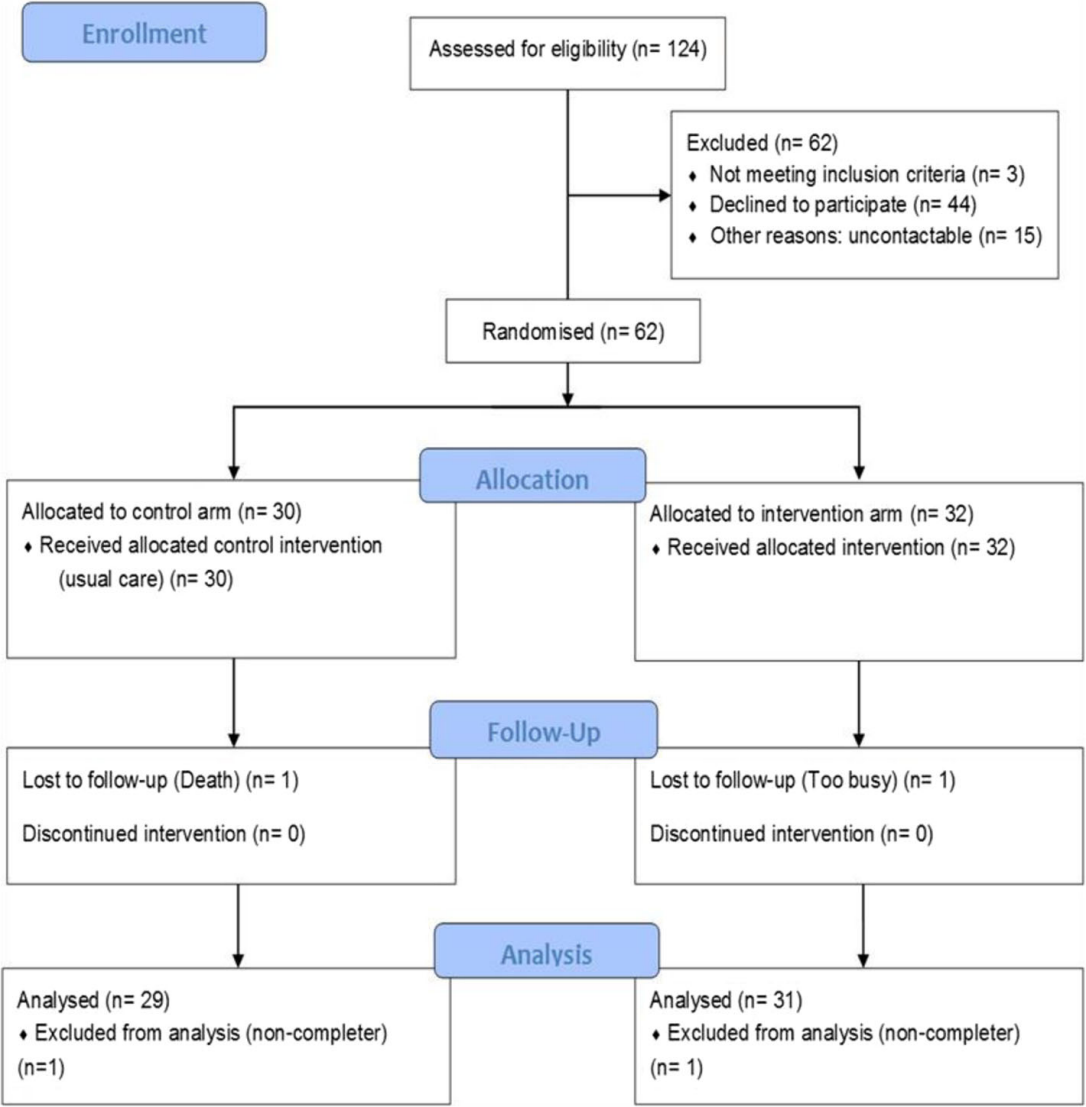
Consolidated Standards of Reporting Trials (CONSORT) flow diagram

Overall, 62 participants were randomised. Recruitment took place over a 16-month period (10th June 2014 through 23rd September 2015), with a participant recruitment rate of 3.9/month. Of the 124 potentially eligible participants approached, 44 declined participation (35%), 15 were uncontactable after attempts to re-contact following receipt of the participant information sheet (12%), and 3 were ineligible (2%). Overall 62 of 124 (50%; 95% CI, 41–59%) of those approached were willing and eligible to enter the study. There were two serious adverse events unrelated to the study, including one death. Two withdrawals from the study were made (one change in personal circumstance, one death). The Consolidated Standards of Reporting Trials (CONSORT) diagram further details participant flow through the study (Fig. 1). The ITT analysis set included all randomised participants (*N* = 62). The study ‘completers’ analysis set included all randomised participants, retaining participants in their randomised allocation groups, who attended their final study visit at 12 weeks post-randomisation (*n* = 60) and was used for analyses of change data.

### Baseline characteristics

Demographic and clinical baseline characteristics were compared across treatment groups descriptively. For these baseline study entry characteristics, data for all participants were included (ITT analysis set; *N* = 62). To describe the population at baseline, features characterising individuals (e.g., age, gender), the nature of their disease (e.g., lung function, severity scores {Bronchiectasis Severity Index [35]}, time since diagnosis, microbiology), and information seeking were recorded (Table 1). Overall more women were recruited than men, which had been anticipated in keeping with the patient demographic. There were approximately equivalent male-to-female ratios in each group. Median age was 65 in both groups. Median FEV_1_ percent predicted values were 75% in the intervention group and 67% in the control group. Most characteristics at baseline were approximately balanced between randomised groups; descriptively, there were more participants in the intervention group who had had bronchiectasis for more than 10 years (17 of 32 [53%] versus 9 of 30 [30%]). The numbers of those who had a relatively new diagnosis (< 1 year) were more even (6 of 30 [20%] in the control group versus 6 of 32 [18%] in the intervention group). The number of patients attending a specialist bronchiectasis clinic formed the majority in both groups.

**Table 1:** Baseline characteristics by allocation group (intention-to-treat analysis set)

	Control group (*n* = 30)	Intervention group (*n* = 32)	Total (*N* = 62)
Gender			
Female	18 (60%)	20 (62.5%)	38 (61%)
Male	12 (40%)	12 (37.5%)	24 (39%)
Age (years)			
Median (range)	65 (34–81)	65 (18–81)	65 (18–81)
FEV_1_ (% predicted)			
Median (range)	75 (21–120)	67 (10–110)	68 (10–120)
BSI score^a^			
Median (range)	6 (2–15)	7 (2–14)	6 (2–15)
BSI severity group			
Mild (score 0–4)	9 (30%)	13 (41%)	22 (35%)
Moderate (score 5–8)	14 (47%)	13 (41%)	27 (44%)
Severe (score > 8)	7 (23%)	6 (19%)	13 (21%)
Time since diagnosis (years)			
Median (range)	6 (0.5–70)	15 (0.25–70)	10 (0.25–70)
> 10	9 (30%)	17 (53%)	26 (42%)
> 5 ≤ 10	5 (17%)	2 (6%)	7 (11%)
> 1 ≤ 5	10 (33%)	6 (19%)	16 (26%)
> 6 months ≤ 1 year	5 (17%)	3 (9%)	8 (13%)
≤ 6 months	1 (3%)	3 (9%)	4 (6%)
Bronchiectasis aetiology			
Idiopathic			
Post-infection	12 (40%)	10 (31%)	22 (35%)
Secondary to chronic asthma/COPD	6 (20%)	11 (34%)	17 (27%)
Immune deficiency associated	7 (23%)	5 (16%)	12 (19%)
Other^b^	2 (7%) 3 (10%)	1 (3%) 5 (16%)	3 (5%) 8 (13%)
Exacerbations per year			
< 3	13 (43%)	10 (31%)	23 (37%)
≥ 3	17 (57%)	22 (69%)	39 (63%)
Use of home intravenous antibiotics			
Y	8 (27%)	15 (47%)	23 (37%)
N	22 (73%)	17 (53%)	39 (63%)
Clinic attended			
Specialist	28 (93%)	30 (94%)	58 (94%)
General	2 (7%)	2 (6%)	4 (6%)
Prior bronchiectasis hospital admissions			
Y	16 (53%)	25 (78%)	41 (66%)
N	14 (47%)	7 (22%)	21 (34%)
Sputum microbiology			
*Pseudomonas aeruginosa*	8 (27%)	9 (28%)	17 (27%)
Other^c^	8 (27%)	11 (34%)	19 (31%)
Not colonised	13 (43%)	11 (34%)	24 (39%)
No samples	1 (3%)	1 (3%)	2 (3%)
Drug treatments			
Azithromycin	10 (33%)	18 (56%)	28 (45%)
Nebulised antibiotics	6 (20%)	3 (9%)	9 (15%)
Devices used to access Internet/resource			
Mobile	2 (7%)	8 (25%)	10 (16%)
Tablet	13 (43%)	10 (31%)	23 (72%)
PC/laptop	28 (93%)	28 (88%)	56 (90%)
No access	0	1 (3%)	1 (2%)
Previous bronchiectasis information seeking			
None	9 (30%)	5 (16%)	14 (23%)
Paper	9 (30%)	14 (44%)	23 (37%)
Online	14 (47%)	16 (50%)	30 (48%)
In person	4 (13%)	3 (9%)	7 (11%)

*Abbreviations: BSI* Bronchiectasis Severity Index, *COPD* chronic obstructive pulmonary disease, *FEV*_*1*_ forced expiratory volume in 1 s, *PC* personal computer

^a^Newcastle Bronchiectasis Severity Index, computed tomographic scoring not included

^b^Pink disease, rheumatoid arthritis, Marfan syndrome, connective tissue disease, granulomatosis with polyangiitis

^c^*Haemophilus influenzae*, *Klebsiella* spp., *Staphylococcus aureus*, *Serratia marcescens*, *Moraxella catarrhalis*, *Escherichia coli*

### Feasibility outcome measures

Sixty-two of 124 (50%; 95% CI, 41–59%) potentially eligible participants were consented and entered the study. A projected participant recruitment rate of two or three per month was surpassed, with an actual recruitment rate of 3.9/month.

Study completion rates were recorded as a measure of acceptability of the trial design to participants, which formed part of the assessment of feasibility. Two participants did not complete the study (one death in the control arm; one lost to follow-up in the intervention arm). There were therefore 60 (97%) of 62 participants in the ‘completers’ analysis set (Table 2). In the study completers group, all participants completed the three required study visits as per protocol.

**Table 2:** Study completion rates

Outcome measure	Total (*N* = 62)	
	Control group (*n* = 30)	Intervention group (*n* = 32)
Study completers	29/30 (97%; 95% CI, 83–99%)	31/32 (97%; 95% CI, 84–99%)

For the purposes of determining feasibility, the completion rates of all questionnaires and lung function tests were examined. Summaries of complete cases for both validated and unvalidated questionnaires and lung function were tabulated (Additional file 4). The majority of questionnaires had 100% completion rate. QOL-B treatment burden does not generate a score if the participant is receiving no treatments, hence an apparent lower completion rate; this reflects the scoring system rather than a reduced completion rate.

### Health-related and quality of life measures

Numbers of complete cases and descriptive analysis of baseline and change data for all validated questionnaires and lung function tests are compiled in Table 3. Baseline means were similar in both groups for the majority of measures, with the exception of a descriptively higher mean FIS score and a higher mean FEV_1_ percent predicted in the control group. Within both the control group and the intervention group, the mean changes from baseline to 12 weeks are mostly considered small, clinically, with their associated SDs indicating relatively large variation in the change between participants. Descriptively, there were mean changes in outcomes in both the direction of improvement and of worsening in both the control group and the intervention group. No further statistical analyses were carried out, in keeping with the pre-defined SAP.

**Table 3:** Summary of baseline and change data for lung function and validated questionnaires

	No.	Control group (*n* = 29)			No.	Intervention group (*n* = 31)		
		Baseline mean (SD)	Mean change^a^, baseline to 2 weeks (SD)	Mean change, baseline to 12 weeks (SD)		Baseline mean (SD)	Mean change, baseline to 2 weeks (SD)	Mean change, baseline to 12 weeks (SD)
EQ-5D-5L^b^								
Index value	29	0.75 (0.24)	0.01 (0.17)	-0.5 (0.19)	31	0.70 (0.23)	0.06 (0.13)	2.11 (11.7)
VAS	29	68.6 (18.5)	−1.55 (13.0)	0.48 (16.2)	31	65.4 (18.0)	5.97 (12.0)	−3.0 (16.7)
SGRQ^c^								
Symptoms	29	57.6 (23.4)		0.25 (15.0)	30	65.9 (21.3)		−7.89 (25.7)
Activity	27	56.3 (23.5)		–4.85 (13.4)	31	56.1 (28.3)		−4.79 (14.8)
Impacts	29	33.6 (21.5)		0.02 (11.3)	31	32.9 (17.2)		−3.06 (13.0)
Total	27	46.1 (19.9)		−1.53 (8.3)	30	44.9 (19.1)		−2.62 (9.46)
QOL-B^d^								
Physical	29	48.1 (32.3)	2.30 (17.8)	-3.85 (16.7)	30	51.6 (35.1)	3.11 (16.2)	-4.94 (25.2)
Role	29	63.5 (29.3)	0.23 (13.6)	1.32 (17.6)	30	64.9 (25.8)	5.33 (18.7)	2.22 (17.8)
Vitality	29	44.1 (24.0)	–1.53 (18.5)	0.38 (16.9)	30	43.7 (22.4)	4.44 (18.4)	-0.74 (21.0)
Emotion	29	84.0 (17)	1.05 (14.9)	-2.1 (13.0)	30	85.0 (18.6)	4.44 (15.3)	2.22 (10.2)
QOL-B								
Social	29	59.7 (31.2)	-0.077 (22.1)	2.97 (14.7)	30	58.1 (22.8)	5.37 (17.2)	3.14 (19.0)
Treatment burden^e^	21	71.2 (22.7)	–1.06 (16.8)	0.53 (14.7)	22	71.7 (21.6)	2.02 (14.8)	1.01 (14.1)
Health	29	45.0 (25.3)	1.25 (18.2)	-0.10 (13.0)	30	44.0 (24.3)	7.04 (15.8)	3.06 (18.4)
Respiration	28	60.6 (23.7)	3.97 (17.3)	3.6 (16.3)	30	54.3 (22.8)	9.7 (14.6)	6.3 (16.8)
HADS^f^								
A	29	5.69 (3.87)	–0.14 (2.86)	–0.31 (3.11)	31	5.87 (4.01)	−1.32 (2.4)	0.44 (6.6)
D	29	4.52 (3.61)	0.00 (2.38)	0.14 (1.76)	31	3.81 (2.33)	−0.10 (1.7)	2.12 (13.2)
FIS^g^								
Cognitive	29	8.52 (9.44)		2.07 (7.2)	31	7.55 (5.84)		1.26 (5.7)
Physical	29	15.52 (12.3)		−1.69 (5.6)	31	12.9 (8.18)		0.39 (6.9)
Social	29	21.79 (21.95)		−0.59 (8.8)	31	15.7 (13.4)		2.72 (12.9)
Total	29	45.8 (42.5)		−0.21 (18.3)	31	36.2 (26.0)		2.70 (19.7)
FEV_1_ (% predicted)	28	72.6 (26.7)		3.78 (8.61)	30	63.7 (23.8)		−0.78 (10.1)
FEV_1_ (L)	28	1.82 (0.73)		0.08 (0.23)	30	1.64 (0.75)		0.23 (1.15)

*Abbreviations: EQ-5D-5L* 5-level EuroQol 5-dimension quality of life scale, *FEV*_*1*_ forced expiratory volume in 1 s, *FIS* Fatigue Impact Scale, *HADS* Hospital Anxiety and Depression Scale, *QOL-B* Quality of Life–Bronchiectasis, *SGRQ* St George’s Respiratory Questionnaire, *VAS* visual analogue scale

^a^A negative change indicates a numerical fall, on average, from baseline to 12 weeks

^b^EuroQol-5D-5L: self-reported health status (VAS range, 0–100%)

^c^St George’s Respiratory Questionnaire: scores are a percentage of overall impairment; 100 represents worst possible health status, and 0 indicates best possible health status

^d^Quality of Life – Bronchiectasis: scored 0–100, higher score = better quality of life

^e^Treatment burden is not scored if participant is receiving no treatment, hence apparent lower *n* value

^f^Hospital Anxiety and Depression Scale: anxiety 0–21, depression 0–21; scores 11+ are significant

^g^Fatigue Impact Scale: cognitive, 0–40; physical, 0–40; social, 0–80; total, 0–160. Higher score = bigger impact of fatigue

### Bronchiectasis knowledge questionnaire

The BKQ is an unvalidated questionnaire consisting of 26 statements detailing understanding of bronchiectasis and its management. Fifteen of the statements ask for a self-grading of understanding using a Likert scale, and 11 ask for a true-or-false response (Additional file 3). A summary of all question responses is tabulated and presented as an appendix due to the volume of data (Additional file 5). BKQ completion rates were high. Descriptively, a greater percentage of participants reported understanding aspects of their condition ‘very well’ by visit 3 for all 15 of the Likert scale statements in the intervention group and for 8 of 15 statements in the control group. Because the BKQ is not validated and in keeping with the pre-defined SAP, the results of the responses to this questionnaire are only reported descriptively. No statistical significance has been attached to this apparent increase in understanding amongst the group receiving the educational intervention in this feasibility study.

### Resource evaluation

The RSQ was administered only to participants in the intervention group. Twenty-seven (84%) of 32 reported using the information provided, 25 (93%) of 27 of users found it useful, particularly the video content. More than 80% thought that the information was easy to use, covered the topics they wanted, and that the right amount was given. By visit 3, 18 (64%) of 28 felt that they were more able to manage their bronchiectasis (Table 4). Feedback about the resource from the RSQ is compiled in Tables 5 and 6.

**Table 4:** Resource Satisfaction Questionnaire review of information overall

	No. completing questionnaire (*n* = 31)	Agree, no. (%)	Neutral, no. (%)	Disagree, no. (%)
1. I found the information useful.				
V2	27	25 (93)	1 (4)	1 (4)
V3	28	26 (93)	1 (4)	1 (4)
2. My knowledge about my condition has improved.				
V2	27	22 (81)	4 (15)	1 (4)
V3	28	18 (64)	8 (29)	2 (7)
3. I feel more able to manage my condition.				
V2	27	13 (48)	12 (44)	2 (7)
V3	28	18 (64)	8 (29)	2 (7)
4. The information provided was easy to understand.				
V2	27	26 (96)	0	1 (4)
V3	28	25 (89)	1 (4)	2 (7)
5. The right amount of information was given.				
V2	27	24 (89)	2 (7)	1 (4)
V3	28	23 (82)	4 (14)	1 (4)
6. The things I wanted to know about were covered.				
V2	27	25 (93)	2 (7)	0
V3	28	24 (86)	2 (7)	2 (7)
7. My partner/family member/friend used the information.				
V2	27	11 (41)	3 (11)	13 (48)
V3	27	15 (56)	4 (15)	8 (30)

**Table 5:** Resource Satisfaction Questionnaire free-text positive comments

	Positive comments
Regarding the use of video	Enjoyed watching the video of patients
	Video clips helpful
	Liked patient video clips
	Found videos comforting
	Found videos of patients doing nebulisers useful
	The videos were good
	Confirmation of own self-management technique helpful also seeing other patients, same problems helpful
Regarding the website	I will use the website in future especially as I will be out of the country for 2 months.
	Website excellent
General overall views	Quite happy with resources provided
	Everything was clear and easy to understand
	Thought good and clear, language good, easy to understand
	Well balanced
	Well done
	I can never have enough information.
Regarding use by family	My youngest son has a better understanding of bronchiectasis.

**Table 6:** Resource Satisfaction Questionnaire free-text suggestions for improvement

	Problems or suggestions
Regarding the use of video	Would like more case studies
	A patient sharing how to use the nebuliser would be more helpful than a technician showing how it works.
	More patient stories
Regarding the website	Problems getting on to Internet site
	Unable to access website
	When scrolling down things disappeared but did come back.
	Login email + password could be clearer
	Patient unable to access website due to invalid password Advice sought from research staff
	Could not access web on my computer
	Could not get on website: password
General overall views	I have not used the information provided due to hospital stay and work commitments.
	I find that now the information has stopped for me. I.e., there is nothing new to read about.
Regarding use by family	Family members not interested, just want me well, quite embarrassed about emotion and keep quiet about my condition.

### Focus group and web analytics

In total, 11 people attended the end-of-study focus group, comprising 8 patient participants and 3 carers, male and female, from both the control and intervention groups. One participant had no Internet access and had elected to enter the study with the PDF version of the site. Participants had a range of ages and times since diagnosis. Participants reported not having found the trial burdensome. Regarding the content of the information resource, participants commented that they had learnt new things and thought it was a good resource for learning about how to find more information on bronchiectasis. The website received favourable reviews, in particular in comparison to other available resources, as this carer stated:

‘To answer your question about the website, because I’ve looked at other things, I think it’s clear, concise.… It’s easier to understand.… The thing that’s good about it you’ve got a carer’s perspective, you’ve got patient perspective, consultant and so on.’ Arthur, 71yrs

They qualified their opinion by explaining that they had seen other resources and explained that they liked the fact that this information resource is both patient- and carer-oriented. Further positive features of the website that were identified were the videos, that you cannot mislay it like you could a leaflet, and that it is easier to update.

Basic web analytics were performed using Google Analytics. Over the duration of the study, there were 7553 sessions of 6456 users, with over 20,000 attempted page views. The large number of attempted page views was due to the fact that the website could be found using search engines but could not be accessed beyond the home page without a password that was a unique code given to each participant in the intervention group. The ‘bounce’ rate (number of users leaving the site without looking beyond the entry page) therefore was high, because only permitted users could access the site. Pages about diet and lifestyle advice were the most popular, with ‘learning about prognosis’; ‘getting a diagnosis’; ‘Why have I got bronchiectasis?’; ‘What symptoms I might get?’; and ‘Who might I need to see?’ also being very popular. Although these data do not accurately describe the use of the site by individual participants, identifying topics of greatest interest to those searching for information about bronchiectasis is of value.

## Discussion

The BRIEF study demonstrated it was feasible to recruit patients into a trial concerning patient education in bronchiectasis. Pre-defined feasibility outcomes were met. Willingness to enter the trial was within predicted numbers, and the recruitment rate was higher than expected. Study design was acceptable as judged by study completion rates, qualitative feedback in the end-of-study focus group, and the completion of study forms as per protocol. Many of the questionnaires used achieved 100% completion rates. The postal questionnaire completion rates were in excess of published average postal questionnaire return rates of 65% [36]. Data quality was exemplary, with very few missing data or errors.

On the basis of these findings, it can be concluded that this study was feasible to conduct. This is key when designing a definitive, larger, multicentre trial based on this study design; yet, there are additional points to consider when moving from pilot to definitive studies [37, 38]. Specifically for the BRIEF study, points to consider for further planning would include the ability to recruit from different centres, planning required follow-up time, retention rates with a longer study follow-up time, and changes to study design to improve chances of recruiting adequate numbers. Planning a definitive trial with an adaptive design would allow for re-assessment of required sample size during recruitment and data collection. For outcomes such as frequency of exacerbations, a much longer follow-up period of at least 1 year would be required, given the likely number of exacerbations per year and the seasonal variation that can occur [39]. Based on the findings of the BRIEF study, this study design is acceptable and feasible to conduct and could be easily adapted to plan a future definitive trial.

There were some apparent differences (descriptively) at baseline between the groups, such as time since diagnosis. Time since diagnosis may influence the type of information needs a person may have. During prior interviews, however, it was apparent that although some information needs do change over time, many unanswered questions were common to those who had both new and long-standing diagnoses [26]. To avoid this being a potential confounder, stratification by time since diagnosis could be considered when planning a full trial. With larger numbers in a definitive trial, however, it would be anticipated that the balance between arms for potential confounders would even out.

Feedback in the RSQ identified that participants used the resource (84%), and 93% of users found it useful. Users highlighted their like of the video clips within the website, particularly those depicting other patients’ stories. Website access or ‘logins’ had been problematic for some. It was unclear whether this was an issue with the website or the users’ devices, but this is clearly an area for improvement. By using the data obtained via website analysis, numbers accessing the site and most popular webpages were identified. Demand for access to the site was shown to be high worldwide, confirming patients want to be able to access credible bronchiectasis information resources. This web analysis facilitated obtaining feedback about the resource and re-confirming prior qualitative findings. This is the first trial of a bronchiectasis information resource that has been based on qualitative exploration and understanding of users’ needs and experiences. Patients and carers were involved in the study and intervention design from the outset, in addition to an independent user representative. A study protocol was produced in keeping with the Medical Research Council guidelines for evaluating complex interventions [40], and this was published and made openly accessible [27]. Findings have been reported in keeping with the CONSORT guidelines [31, 41], and a pre-defined SAP was used when analysing data. As with any study, however, there are some limitations to consider.

The first limitation concerns blinding. This was a feasibility study, with limited funding and a sample size of 62. Due to the nature of the intervention, participants could not be blinded to whether they were receiving the resource to use whilst in the study. As the research staff conducting study visits needed to provide access details and support to those within the intervention group, they too were unblinded in order to ensure data were analysed without bias and not influenced during analysis, and a pre-defined SAP was written with advice from a statistician prior to data analysis [27].

At the time of study commencement, there was no gold standard for objectively measuring what constitutes an exacerbation in bronchiectasis. More recently, however, a consensus has been reported for the definition to be used in clinical research [42]. A future study would use this definition. The BRIEF study used participant recollection and case note review to identify exacerbations. Patient reviewers of the initial study design raised concerns about the potential burden of a daily symptom diary. Hence, we opted to ask participants to record such details on a monthly basis. In a definitive trial of this intervention, however, improvements in self-management and consequently disease stability would need to be assessed. When adapting this study design for a future trial, a protocol definition of an exacerbation and more rigorous reporting of events would be preferable. This could include, for example, electronic symptom diaries.

Although participants were recruited from all respiratory clinics within the trust, at two sites, 58 (94%) of 62 were recruited from the specialist bronchiectasis clinic. The aim was to have a participant group who had different clinical experiences and differing information provision. Those attending a specialist clinic would potentially have access to a level of information beyond that expected in a general respiratory clinic. This is not necessarily the case. Arguably, however, which clinic the participant attended, or prior information provision, could be considered as a stratification variable during randomisation for a definitive trial if thought to be a potential confounding factor. Other related factors that could potentially influence outcomes or the impact of an educational intervention could also be considered as stratification variables for a full trial. Examples could include time since diagnosis (which could influence the nature of information required and prior opportunities to access information), access to the Internet (which could affect formats of resource able to fully engage with) and education or health literacy levels (which could influence both types of resources required and ability to engage with and understand them).

Accessing additional information resources during the BRIEF study was not restricted. The significant difference in information provision for the two groups within the trial was the novel information resource. The ability to monitor additional seeking or provision of information is limited and really has to be accepted as a variable which is not practicably controllable and reflects ‘usual’ patient practices. At study conclusion, all those who had taken part in the study (including those in the control group) were given access to the resource indefinitely.

Numbers of courses of antibiotics would be important information to collect within a definitive trial, though it would be difficult to use as a primary outcome measure. Because the intervention would aim to improve participants’ recognition and management of exacerbations, this could lead to an increase in number of antibiotic courses taken (if a participant had previously been under-recognising treatment requirements); yet, for others, it may lead to a reduction (if they had previously been reacting to ‘normal’ variations in symptoms unnecessarily). Measuring change in number of courses of antibiotics therefore would be difficult to interpret with meaning. It is likely that exacerbation rate, along with a primary outcome measure of unscheduled healthcare visits (representing unpredicted and unmanaged exacerbations), would be the most accurate measure of improvements in self-management and health outcomes. This would be in conjunction with assessing knowledge and understanding of bronchiectasis. In order to more accurately report this, further work would need to be done to improve on and validate the knowledge questionnaires used within the BRIEF study.

Despite the limitations of the BRIEF study, the design had a number of strengths, and it was conducted successfully. The majority of the highlighted limitations are factors to consider for a definitive trial based on this feasibility study. Resource evaluation and qualitative data added to the usefulness of this study by informing refinements to both the resource and trial design. For this feasibility study design to be adapted for a future definitive trial, changes would need to be made as discussed, and an initial sample size calculated using the data presented.

## Conclusions

The BRIEF study was feasible to conduct and indicates that a future, multicentre study based on this trial design to evaluate the impact of this resource on patient understanding, self-management, health outcomes and health service use would be feasible to conduct.

## Supplementary information

**Additional file 1.** Resource Satisfaction Questionnaire: unvalidated questionnaire used within the study.

**Additional file 2.** Postal data collection form: used within study.

**Additional file 3.** Bronchiectasis knowledge questionnaire. Unvalidated questionnaire used to assess participant knowledge of bronchiectasis.

**Additional file 4.** Questionnaire completion rates. Table detailing completion rates of study questionnaires.

**Additional file 5.** Bronchiectasis knowledge questionnaire results. Table detailing responses to the BKQ.

## Data Availability

All data generated or analysed during this study are included in this published article and its supplementary information files.

## Abbreviations

BKQ: Bronchiectasis knowledge questionnaire
BRIEF: BRonchiectasis Information and Education Feasibility study
BSI: Bronchiectasis Severity Index
BTS: British Thoracic Society
CONSORT: Consolidated Standards of Reporting Trials
COPD: Chronic obstructive pulmonary disease
EQ-5D: EuroQol five-dimension quality of life scale
EQ-5D-5L: 5-Level EuroQol 5-dimension quality of life scale
FEV_1_: Forced expiratory volume in 1 s
FIS: Fatigue Impact Scale
HADS: Hospital Anxiety and Depression Scale
HRQOL: Health-related quality of life
ITT: Intention to treat
MREC: Main research ethics committee
NIHR: National Institute for Health Research
PI: Principal investigator
QOL-B: Quality of Life–Bronchiectasis
RCT: Randomised controlled trial
RSQ: Resource Satisfaction Questionnaire
SAP: Statistical analysis plan
SGRQ: St George’s Respiratory Questionnaire
VAS: Visual analogue scale
