# Supplementary material for: Bronchiectasis Information and Education: a randomised, controlled feasibility trial
Source: Trials. 2020 Apr 15;21:331. doi: 10.1186/s13063-020-4134-5 (PMC7158127; doi:10.1186/s13063-020-4134-5)
Supplement: Supplementary file 1 — Additional file 1. Resource Satisfaction Questionnaire: unvalidated questionnaire used within the study. [file 13063_2020_4134_MOESM1_ESM.docx]

**BRIEF Resource satisfaction questionnaire**

Please answer the questions below about your use of the provided information. There are no right or wrong answers and your responses are confidential. Thank you for taking the time to complete this questionnaire.

**I used the information provided: YES/NO**

**(Please circle. If yes answer questions below, if no go to next page)**

|  | **Strongly disagree** | **Disagree** | **Neither disagree nor agree** | **Agree** | **Strongly agree** |
| --- | --- | --- | --- | --- | --- |
| **I found the information useful** |  |  |  |  |  |
| **My knowledge about my condition has improved** |  |  |  |  |  |
| **I feel more able to manage my condition** |  |  |  |  |  |
| **The information provided was easy to understand** |  |  |  |  |  |
| **The right amount of information was given** |  |  |  |  |  |
| **The things I wanted to know about were covered** |  |  |  |  |  |
| **My partner/family member/friend used the information** |  |  |  |  |  |

Please use this space to add any other comments and suggestions for improvement

**About the website**

**I used the website: YES/NO (Please circle. If yes answer questions below, if no go to next page)**

|  | **Strongly disagree** | **Disagree** | **Neither disagree nor agree** | **Agree** | **Strongly agree** |
| --- | --- | --- | --- | --- | --- |
| **I used the website more than the overview booklet** |  |  |  |  |  |
| **The website was my preferred version of the information** |  |  |  |  |  |
| **It was easy to find the sections I wanted to look at** |  |  |  |  |  |
| **I only looked at certain sections** |  |  |  |  |  |
| **I looked at all of the website** |  |  |  |  |  |
| **The login procedure was easy to use** |  |  |  |  |  |
| **I found the video clips helpful** |  |  |  |  |  |
| **I found the diagrams helpful** |  |  |  |  |  |
| **My partner/family member/friend used the website** |  |  |  |  |  |

Please use this space to add any other comments and suggestions for improvement

**About the overview booklet provided**

**I used the overview booklet: YES/NO (Please circle. If yes answer questions below, if no go to next page)**

|  | **Strongly disagree** | **Disagree** | **Neither disagree nor agree** | **Agree** | **Strongly agree** |
| --- | --- | --- | --- | --- | --- |
| **I used the overview booklet more than the website** |  |  |  |  |  |
| **The overview booklet was my preferred version of the information** |  |  |  |  |  |
| **It was easy to find the sections I wanted to look at** |  |  |  |  |  |
| **I only looked at certain sections** |  |  |  |  |  |
| **I looked at the whole overview booklet** |  |  |  |  |  |
| **I found the diagrams helpful** |  |  |  |  |  |
| **The text was easy to read** |  |  |  |  |  |
| **My partner/family member/friend used the overview booklet** |  |  |  |  |  |

Please use this space to add any other comments and suggestions for improvement

**About the downloaded full booklet available on the website (or those who have the PDF in place of internet access at study start)**

**I downloaded the full booklet: YES/NO (Please circle. If yes answer questions below, if no go to next page)**

**I printed out the booklet: YES / NO**

**I received the full booklet in place of the website as I do not use the internet: YES/NO (circle)**

|  | **Strongly disagree** | **Disagree** | **Neither disagree nor agree** | **Agree** | **Strongly agree** |
| --- | --- | --- | --- | --- | --- |
| **I used the full booklet more than the website or overview booklet** |  |  |  |  |  |
| **The full booklet was my preferred version of the information** |  |  |  |  |  |
| **It was easy to find the sections I wanted to look at** |  |  |  |  |  |
| **I only looked at certain sections** |  |  |  |  |  |
| **I looked at the whole full booklet** |  |  |  |  |  |
| **I found the diagrams helpful** |  |  |  |  |  |
| **The text was easy to read** |  |  |  |  |  |
| **My partner/family member/friend used the full booklet** |  |  |  |  |  |

Please use this space to add any other comments and suggestions for improvement
