# Supplementary material for: Bronchiectasis Information and Education: a randomised, controlled feasibility trial
Source: Trials. 2020 Apr 15;21:331. doi: 10.1186/s13063-020-4134-5 (PMC7158127; doi:10.1186/s13063-020-4134-5)
Supplement: Supplementary file 2 — Additional file 2. Postal data collection form: used within study. [file 13063_2020_4134_MOESM2_ESM.docx]

**BRIEF Study Bronchiectasis Knowledge Questionnaire**

Please indicate how well you feel you understand the following:

|  | **Very well** | **Quite well** | **Not very well** | **Not at all well** |
| --- | --- | --- | --- | --- |
| I understand what bronchiectasis is |  |  |  |  |
| I understand why bronchiectasis gives me the symptoms that I have |  |  |  |  |
| I understand the prognosis or long term effects bronchiectasis might have |  |  |  |  |
| I understand what can cause bronchiectasis |  |  |  |  |
| I understand what the bronchiectasis medications I take are for |  |  |  |  |
| I know what signs might tell me I am having a bronchiectasis flare up and need antibiotics |  |  |  |  |
| I know what to do when I have a bronchiectasis flare up |  |  |  |  |
| I understand what extra things I can do to help myself look after my bronchiectasis |  |  |  |  |
| I know who to go to when I need help or advice about bronchiectasis |  |  |  |  |
| I understand my bronchiectasis clinic letter from the doctor |  |  |  |  |
| I know where to find more information on bronchiectasis |  |  |  |  |
| I know who I might see at the hospital and why |  |  |  |  |
| I know how to pronounce bronchiectasis |  |  |  |  |
| I can explain about my bronchiectasis to others |  |  |  |  |
| I feel I can cope and live with my bronchiectasis |  |  |  |  |

Please indicate what you think about the following statements:

|  | **True** | **False** | **Don’t know** |
| --- | --- | --- | --- |
| Bronchiectasis is always caused by smoking |  |  |  |
| Bronchiectasis is the same as Chronic Obstructive Pulmonary Disease (COPD) |  |  |  |
| Bronchiectasis **cannot** be cured in most cases but can be managed or controlled |  |  |  |
| Bronchiectasis can be cured in most cases |  |  |  |
| Fatigue or extreme tiredness can be a symptom of bronchiectasis |  |  |  |
| Antibiotics for bronchiectasis chest infections should be taken for 5 days and then stopped |  |  |  |
| Antibiotics are **always** needed if I am coughing phlegm |  |  |  |
| Chest clearance, breathing exercises or physio are only required during an infection |  |  |  |
| It is recommended I should have a flu jab every year |  |  |  |
| I should not exercise if I have bronchiectasis |  |  |  |
| Patients with bronchiectasis sometimes cough up blood when they have a chest infection |  |  |  |
