# Supplementary material for: Bronchiectasis Information and Education: a randomised, controlled feasibility trial
Source: Trials. 2020 Apr 15;21:331. doi: 10.1186/s13063-020-4134-5 (PMC7158127; doi:10.1186/s13063-020-4134-5)
Supplement: Supplementary file 3 — Additional file 3. Bronchiectasis knowledge questionnaire. Unvalidated questionnaire used to assess participant knowledge of bronchiectasis. [file 13063_2020_4134_MOESM3_ESM.docx]

Bronchiectasis Information and Education Feasibility Study (BRIEF Study)

A randomised controlled trial

**STATISTICAL ANALYSIS PLAN**

Author: Katy Hester

Last modified: 06.10.2016

**Trial details:**

Chief Investigator: Dr Katy Hester

Principal Investigator: Dr Anthony de Soyza

Statistician: Ms Vicky Ryan

Trial Manager: Mr Chris Speed (No longer in post) (NCTU)

ISRCTN Number: ISRCTN84229105

REC Reference: 14/NE/0119

Sponsor: Newcastle upon Tyne Hospitals NHS Foundation Trust

Sponsor Protocol Number: 7005

Funder: NIHR DRF (DRF-2012-05-149)

Study design: A feasibility study for the proposed future BRIE RCT

Study intervention: Arm 1: Novel patient information resource

Arm 2: Usual care

Primary objective: To inform the decision of whether to proceed to the BRIE RCT and whether any refinements to the design or conduct of that trial are warranted.

Secondary objective: To evaluate and further refine the patient information resource and collect information on patient preferences.

Specific Objectives:

- To assess participants’ willingness to enter the trial
- To gauge participants’ acceptability of study design
- To study length of time required to complete recruitment
- To study retention rate and completion of required study forms
- To evaluate the intervention and experience of trial involvement

Primary outcome: The confirmation or otherwise that the study site will be able to identify, recruit and retain the required number of eligible participants. The acceptability of the intervention (as manifested through recruitment and retention levels), the feasibility and acceptability of the data collection tools (completion rates and quality of data) and clinical data to calculate the sample size for the BRIE RCT. Production of protocols for the full trial.

Secondary outcome: To modify and refine the patient information resource and its delivery based on quantitative and qualitative data.

Specific outcome measures:

- Participants’ willingness to enter the trial (consented participant to eligible participants approached ratio).
- Participants’ acceptability of study design (as measured by the completion rate of participants in each randomised group).
- Participant recruitment rate (as measured by the number of patients randomised divided by the length of the recruitment period). The recruitment period runs from the date that recruitment opened to the date of the last randomisation.
- Completion of forms.
- Evaluation of resource and trial experience to include ‘compliance with’ (use of) intervention.

Study site: Newcastle upon Tyne Hospitals NHS Trust – Freeman and RVI sites for recruitment, Freeman only for study visits.

Sample size required: 60 (30 in each arm) plus up to 10 for focus groups to discuss resource and trial.

Study duration: 28 months. A patient is within the main study for 3 months (calculated at 12 weeks or 84 days).

Protocol approval and amendment dates:

| **Protocol version** | **Amendment** | **Details** | **Approved Date** |
| --- | --- | --- | --- |
| V1.0, dated 23/04/2014 | Original submission | 14/NE/0119 | 02/05/2014 |
| V1.1, dated 27/05/2014 | As per ethical approval committee meeting | To include those without internet access | 29/05/2014 |
| V1.2,  dated 11/06/2015 | Minor protocol changes | Continued access to resource after study completion and access for control group after study completion | 16/06/2015 |
| V1.3  Dated 13/08/2015 | Minor protocol Changes | No FEV1 required at V1 if recorded in past 3 months | 26/08/2015 |

**Introduction**

The trial protocol describes the method of data collection and the main features of the analysis. This document describes, in more detail, the proposed strategy for the statistical analysis and presentation of data collected for this trial, guiding the final analyses.

Both the ICH Guidance on Statistical Principles for Clinical Trials (ICH E9) and the Revised CONSORT Statement for Reporting Randomized Trials recommend that all analyses should be planned and outlined in a statistical analysis plan prior to the unblinding of the data so as to avoid any post hoc decisions which may affect the interpretation of the statistical analyses. The CONSORT statement also recommends that when writing research papers authors should specify whether analyses were planned or suggested by the data – planned analyses have greater credibility and are in line with Good Clinical Practice. The BRIEF study was not blinded due to the nature of the intervention making this impractical. The same principles have been applied for the purposes of analysis however.

1. **Data validation**

Trial data has been entered manually into the BRIEF study database by the data managers at the William Leech Centre for Lung Research from where the study was conducted. All data is originally hand written into each study record folder and then entered into an excel database subsequently.

The following checks will be carried out before any analysis:

- treatment arm allocation as recorded in the randomisation log checked against that recorded in the CRF
- randomisation stratification variable as recorded in the randomisation log, checked against Visit 1 CRF
- Age at randomisation ≥18 years (from randomisation log and CRF visit 1)
- examine all dates to check timings and compliance with the protocol.

1. **Recruitment**

The BRIEF study aimed to randomise 60 patients.

Study open dates run from June 2014 until Sep 2016.

Recruitment is expected to be over 28 months and projected patient accrual 2-3 patients/month.

A subsequent maximum of 10 patients and carers will be recruited (but not randomised) to a focus group that aims to discuss the resource and the trial process. This data will be purely qualitative in nature and is not included within this analysis plan.

**2.1 Recruitment summary:**

Non completers (n=)

Reason:

Non completers (n=)

Reason:

Completed study (n=)

Completed study (n=)

Control arm (n=)

Intervention arm (n=)

PDF only entry (n=)

Consented and randomized (n=)

Screen fails/

not interested

in taking part

Assessed for eligibility (n=)

**2.2** **Plot of cumulative number of patients randomised: actual and predicted**

*Plot will be inserted here*

1. **Distribution of patients by randomisation strata**

Patients will be randomised using random permuted blocks within strata.

Randomisation is stratified according to gender.

Randomisation is to be performed by the research staff using the Newcastle University online randomisation system.

**3.1** **Table of the distribution of patients by randomisation strata**

| **Strata** | **Control**  **Group**  ***n=*** | **Intervention**  **Group**  ***n*=** | **Total**  ***n* =** |  |
| --- | --- | --- | --- | --- |
| Male | |  |  |  |
| Female | |  |  |  |
|  | |  |  |  |
|  | |  |  |  |

**4. Ineligible participants**

The number of ineligible patients and reasons for ineligibility will be reported.

**5. Baseline patient characteristics**

Demographic and clinical baseline characteristics and trial stratification factors at randomisation will be compared across treatment groups descriptively. Descriptive statistics will be tabulated by treatment group and overall.

No significance testing will be carried out due to the randomised nature of the study^1,2,3^.

Formal tests will not be carried out but any imbalances will be explored further (see analyses of outcome measures below).

**Baseline characteristics, by allocation group (*n*=).**

|  | **Control group**  ***n*=** | **Intervention**  **group**  ***n*=** | **Total**  ***n* =** |
| --- | --- | --- | --- |
| **Gender**  Female  Male | x (%)  x (%) | x (%)  x (%) | x (%)  x (%) |
|  |  |  |  |
| **Age (years)**  Median (range) | m (range) | m (range) | m (range) |
|  |  |  |  |
| **FEV1 (% predicted)**  Median (range) |  |  |  |
|  |  |  |  |
| **BSI score***  Median (range)  **BSI Severity group**  Mild (score 0-4)  Moderate (score 5-8)  Severe (score >8) |  |  |  |
| **Time since diagnosis (years)**  Median (range)  > 10  > 5 ≤ 10  >1 ≤ 5  >6 months ≤1 year  ≤6 months |  |  |  |
| **Bronchiectasis aetiology**  Idiopathic  Post-infection  Secondary to chronic asthma/COPD  Immune deficiency associated  Other** |  |  |  |
|  |  |  |  |
| **Exacerbations per year**  <3  ≥3 |  |  |  |
|  |  |  |  |
| **Use of home IVs**  Y  N  **Clinic attended**  Specialist  General  **Prior bronchiectasis hospital admissions**  Y  N  **Sputum microbiology**  Pseudomonas aeruginosa  Other***  Not colonised  No samples  **Drug treatments**  Azithromycin  Nebulised antibiotics  **Devices used to access internet/resource**  Mobile  Tablet  PC/laptop  No access  **Previous bronchiectasis information seeking**  None  Paper  Online  In person |  |  |  |

*^[[1]](#footnote-1)^*

**6. Summary of AEs, SAEs, withdrawals and loss to follow-up**

**6.1 Chronological listing of SAEs**:

| **group allocation** | **Date of initial report** | **SAE Description** | **Onset Date** | **SAE reason** | **Outcome of SAE** |
| --- | --- | --- | --- | --- | --- |
|  |  |  |  |  |  |

**6.2 Chronological listing of withdrawals**:

| **group allocation** | **Randomisation date** | **Withdrawal date** | **Days in the study** | **Type of withdrawal** | **Reason/comment** |
| --- | --- | --- | --- | --- | --- |
|  |  |  |  |  |  |

**6.3 Chronological listing of patients lost to follow up:**

If *x* withdrawals listed in Section 6.2 agreed for their data up to the point of withdrawal to be used for study purposes, there data will be used up until point of withdrawal, but these patients will not be followed up further within the study.

**7. Definition of analysis group**

Statistical analyses will be based on the intention to treat principle with analysis groups based on the groups allocated at randomisation and all randomised patients being included in the analysis. Missing data due to participant loss to follow-up or non-completion of study visits or questionnaires will not be imputed and therefore the analyses of change data will be for complete cases.

Due to the nature of the intervention adherence to protocol was not assessed.

**8.** **Analysis of outcome measures**

**Primary outcome measures:**

- Participants’ willingness to enter the trial (consented participant to potentially eligible participants approached ratio).
- Participants’ acceptability of study design (as measured by the completion rate of participants in each randomised group).
- Participant recruitment rate (as measured by the number of patients randomised divided by the length of the recruitment period). The recruitment period runs from the date that recruitment opened to the date of the last randomisation.
- Participant completion of required study forms and visits

**Secondary outcome measures:**

- Resource satisfaction questionnaire
- Recorded use of resource and alternative information seeking
- QOL-B
- SGRQ
- HADS
- FIS
- EQ-5D
- Number of unscheduled visits to primary or secondary care
- Exacerbation frequency
- FEV1
- Knowledge of condition and management questionnaire

In accordance with recommendations for the analysis of feasibility studies^4,5^ (where a formal power calculation is not carried out) the data analysis will be descriptive and statistical comparisons between the randomisation groups will not be undertaken.

For the primary outcome measures all proportions/rates will be calculated as defined.

Example table:

| **Outcome measure** | **(n= )** | |
| --- | --- | --- |
|  | **Control group**  ***n*=** | **Intervention group**  ***n*=** |
| Percent remaining in the trial up to week 12 | x/n (%) | x/n (%) |

All summaries will be interpreted cautiously because of the size of the study and the possible imbalance in pre-randomisation baseline covariates.

Secondary outcome measures will be described at baseline and the change from baseline to 12 weeks will be summarised (and reported as mean (sd) or median (IQR) depending on the distribution of the data).

For missing data, published guidelines will be followed for validated questionnaires and otherwise generic rules (such as ‘rule of halves’) which are recommended in the literature will be described and employed. The details of individual questionnaires will be described individually.

| **Variable** | **n*** | **Control group *n*=** | | | **n*** | **Intervention group *n*=** | | |
| --- | --- | --- | --- | --- | --- | --- | --- | --- |
|  |  | **Baseline**  **mean (sd)** | **mean change****  **baseline to**  **2 weeks (sd)** | **mean change****  **baseline to**  **12 weeks (sd)** |  | **Baseline**  **mean (sd)** | **mean change****  **baseline to**  **2 weeks (sd)** | **mean change****  **baseline to**  **12 weeks (sd)** |
| **RSQ** |  |  |  |  |  |  |  |  |
| **BKQ** |  |  |  |  |  |  |  |  |
| **EQ-5D** |  |  |  |  |  |  |  |  |
| **SGRQ** |  |  |  |  |  |  |  |  |
| **QOL-B** |  |  |  |  |  |  |  |  |
| **HADS** |  |  |  |  |  |  |  |  |
| **FIS** |  |  |  |  |  |  |  |  |
| **FEV1** |  |  |  |  |  |  |  |  |
| **Recorded use of resource** |  |  |  |  |  |  |  |  |
| **Recorded information seeking** |  |  |  |  |  |  |  |  |
| **Exacerbation frequency** |  |  |  |  |  |  |  |  |
| **Number of unscheduled presentations** |  |  |  |  |  |  |  |  |

*n=number of participants with all three measures

**A negative change indicates a fall on average from baseline to 12 weeks

**8.1 Treatment** **comparisons**

The study has not been designed to make treatment comparisons or draw inferences and as such no formal statistical testing will be performed.

**8.2 Informing future studies**

**References:**

1. Moher D, Hopewell S, Shultz KF, et al, CONSORT 2010 Explanation and Elaboration: updated guidelines for reporting parallel group randomised trials. *BMJ* 2010;340:c869 doi: 10.1136/bmj.c869
2. Altman DG, Comparability of randomised groups. *Statistician* 1985;34:125-36.
3. Roberts C and Torgerson DJ, Baseline imbalance in randomised controlled trials. *BMJ* 1999;319:185
4. Lancaster GA, Dodd S, Williamson PR, et al. Design and analysis of pilot studies:

recommendations for good practice. *J Eval Clin Pract* 2004;10:307–12.

1. Thabane L, Ma J, Chu R, et al. A tutorial on pilot studies: the what, why and how.

*BMC Med Res Methodol* 2010;10:1.

1. * Newcastle Bronchiectasis Severity Index, CT scoring not included

   ** Pink’s Disease, Rheumatoid arthritis, Marfan’s Syndrome, Connective Tissue Disease, Wegener’s Granulomatosis

   *** Haemophilus Influenzae, Klebsiella, Staphylococcus aureus, Serratia marcescens, Maroxella catarrhalis, Escherichia Coli. [↑](#footnote-ref-1)
