# Supplementary material for: Bronchiectasis Information and Education: a randomised, controlled feasibility trial
Source: Trials. 2020 Apr 15;21:331. doi: 10.1186/s13063-020-4134-5 (PMC7158127; doi:10.1186/s13063-020-4134-5)
Supplement: Supplementary file 4 — Additional file 4. Questionnaire completion rates. Table detailing completion rates of study questionnaires. [file 13063_2020_4134_MOESM4_ESM.docx]

Summary of number of complete questionnaires and lung function at each study visit by randomisation group.

| **Outcome measure** | **Control group complete cases (n=29)**  **Number (% rounded)** | | | | **Intervention group complete cases (n=31)**  **Number (% rounded)** | | | |
| --- | --- | --- | --- | --- | --- | --- | --- | --- |
|  | **V1** | **V2** | **V3** | **Total complete at all visits** | **V1** | **V2** | **V3** | **Total complete at all visits** |
| **FEV1 % predicted** | 29(100) |  | 28 (97) | 28 (97) | 31(100) |  | 30 (98) | 30 (98) |
| **FIS:-**  **Cognitive**  **Physical**  **Social**  **Total** | 29(100)  29(100)  29(100)  29(100) |  | 29(100)  29(100)  29(100)  29(100) | 29(100)  29(100)  29(100)  29(100) | 31(100)  31(100)  31(100)  31(100) |  | 31(100)  31(100)  31(100)  31(100) | 31(100)  31(100)  31(100)  31(100) |
| **EQ-5D-5L** | 29(100) | 29(100) | 29(100) | 29(100) | 31(100) | 31(100) | 31(100) | 31(100) |
| **HADS** | 29(100) | 29(100) | 29(100) | 29(100) | 31(100) | 31(100) | 31(100) | 31(100) |
| **SGRQ:-**  **Symptoms**  **Activity**  **Impacts**  **Total** | 29(100)  28(97)  29(100)  28(97) |  | 29(100)  28(97)  29(100)  28(97) | 29(100)  27(93)  29(100)  27(93) | 31(100)  31(100)  31(100)  31(100) |  | 30(98)  31(100)  31(100)  30(98) | 30(98)  31(100)  31(100)  30(98) |
| **QOL-B:-**  **Physical**  **Role**  **Vitality**  **Emotion**  **Social**  **Treatment Burden**  **Health**  **Respiration** | 29(100)  29(100)  29(100)  29(100)  29(100)  23(79)  29(100)  29(100) | 29(100)  29(100)  29(100)  29(100)  29(100)  25(86)  29(100)  29(100) | 29(100)  29(100)  29(100)  29(100)  29(100)  26(90)  29(100)  28(97) | 29(100)  29(100)  29(100)  29(100)  29(100)  21(72)  29(100)  28(97) | 31(100)  31(100)  31(100)  31(100)  31(100)  27(87)  31(100)  31(100) | 31(100)  31(100)  31(100)  31(100)  31(100)  26(84)  31(100)  31(100) | 31(100)  31(100)  31(100)  31(100)  31(100)  27(87)  31(100)  31(100) | 31(100)  31(100)  31(100)  31(100)  31(100)  22(71)  31(100)  31(100) |

Summary of completion and return of unvalidated questionnaires.

| Outcome measure | Control group complete cases (n=29)  Number (% rounded) | | | | Intervention group complete cases (n=31) Number (% rounded) | | | |
| --- | --- | --- | --- | --- | --- | --- | --- | --- |
|  | V1 | V2 | V3 | Total completing at all visits | V1 | V2 | V3 | Total completing at all visits |
| RSQ |  |  |  |  |  | 31(100) | 31(100) | 31(100) |
| BKQ | 29(100) | 28(97) | 29(100) | 28(97) | 31(100) | 30(97) | 31(100) | 30(97) |
| Postal | 24(83) | 27(93) | 29(100) | 21(72) | 30(97) | 28(90) | 31(100) | 27(87) |
