# Supplementary material for: Bronchiectasis Information and Education: a randomised, controlled feasibility trial
Source: Trials. 2020 Apr 15;21:331. doi: 10.1186/s13063-020-4134-5 (PMC7158127; doi:10.1186/s13063-020-4134-5)
Supplement: Supplementary file 5 — Additional file 5. Bronchiectasis knowledge questionnaire results. Table detailing responses to the BKQ. [file 13063_2020_4134_MOESM5_ESM.docx]

|  | **n** | **Control group n and (%)** | | | | **n** | **Intervention group n and (%)** | | | |
| --- | --- | --- | --- | --- | --- | --- | --- | --- | --- | --- |
| **Question/Visit** |  | **Not at all well** | **Not very well** | **Quite well** | **Very well** |  | **Not at all well** | **Not very well** | **Quite well** | **Very well** |
| **1. I understand what bronchiectasis is.**  V1  V2  V3 | 29  28  29 | 0  0  0 | 3(10)  3(11)  2(7) | 15(52)  15(54)  17(59) | 11(38)  10(36)  10(34) | 31  30  31 | 1(3)  0  0 | 3(10)  0  0 | 17(55)  11(37)  9(29) | 10(32)  19(63)  22(71) |
| **2. I understand why bronchiectasis gives me the symptoms that I have.**  V1  V2  V3 | 29  28  29 | 0  0  0 | 6(21)  4(14)  2(7) | 15(52)  13(46)  16(55) | 8(28)  11(39)  11(38) | 31  30  31 | 0  0  0 | 6(19)  0  2(6) | 13(42)  12(40)  7(23) | 12(39)  18(60)  22(71) |
| **3. I understand the prognosis or long term effects bronchiectasis might have.**  V1  V2  V3 | 29  28  29 | 2(7)  2(7)  1(3) | 12(41)  8(29)  9(31) | 9(31)  8(29)  10(34) | 6(21)  10(36)  9(31) | 31  30  31 | 2(6)  0  0 | 8(26)  4(13)  2(6) | 11(35)  14(47)  13(42) | 10(32)  12(40)  16(52) |
| **4. I understand what can cause bronchiectasis.**  V1  V2  V3 | 29  28  29 | 1(3)  3(11)  0 | 9(31)  10(36)  8(28) | 14(48)  8(29)  13(45) | 5(17)  7(25)  8(28) | 31  30  31 | 6(19)  1(3)  3(10) | 10(32)  6(20)  4(13) | 8(26)  11(37)  8(26) | 7(23)  12(40)  16(52) |
| **5. I understand what the bronchiectasis medications I take are for.**  V1  V2  V3 | 29  28  28 | 0  0  0 | 0  3(11)  0 | 12(41)  11(39)  13(46) | 17(59)  14(50)  15(54) | 31  30  31 | 0  0  0 | 6(19)  2(7)  1(3) | 12(39)  13(43)  8(26) | 13(42)  15(50)  22(71) |
| **6. I know what signs might tell me I am having a bronchiectasis flare up and need antibiotics.**  V1  V2  V3 | 29  27  29 | 0  0  0 | 4(14)  4(15)  1(3) | 9(31)  11(41)  11(38) | 16(55)  12(43)  17(59) | 31  30  31 | 1(3)  0  0 | 3(10)  2(7)  0 | 12(42)  8(27)  8(26) | 15(48)  20(67)  23(74) |
| **7. I know what to do when I have a bronchiectasis flare up.**  V1  V2  V3 | 29  28  29 | 1(3)  0  0 | 1(3)  1(4)  2(7) | 9(31)  10(36)  9(31) | 18(62)  17(61)  18(62) | 31  30  31 | 1(3)  0  0 | 2(6)  0  0 | 11(35)  7(23)  6(19) | 17(55)  23(77)  25(81) |
| **8. I understand what extra things I can do to help myself look after my bronchiectasis.**  V1  V2  V3 | 29  28  29 | 0  0  0 | 6(21)  3(11)  5(17) | 16(55)  8(29)  10(34) | 7(24)  20(71)  14(48) | 31  30  31 | 1(3)  0  0 | 6(19)  2(7)  2(6) | 11(35)  12(40)  11(35) | 13(42)  16(53)  18(58) |
| **9. I know who to go to when I need help or advice about my bronchiectasis.**  V1  V2  V3 | 29  28  29 | 0  0  0 | 1(3)  0  2(7) | 8(28)  12(43)  6(21) | 20(69)  16(57)  21(72) | 31  30  31 | 0  0  0 | 4(13)  1(3)  0 | 6(19)  7(23)  8(26) | 21(68)  22(73)  23(74) |
| **10. I understand my bronchiectasis clinic letter from the doctor.**  V1  V2  V3 | 29  28  29 | 0  0  0 | 2(7)  0  1(3) | 10(34)  12(43)  11(38) | 17(59)  16(57)  17(59) | 31  29  31 | 0  0  0 | 3(10)  4(14)  0 | 10(32)  7(24)  10(32) | 18(58)  18(62)  21(68) |
| **11. I know where to find more information on bronchiectasis.**  V1  V2  V3 | 29  28  29 | 1(3)  0  0 | 7(24)  3(11)  5(17) | 9(31)  14(50)  10(34) | 12(41)  11(39)  14(48) | 31  30  31 | 1(3)  0  0 | 11(35)  0  1(3) | 10(32)  10(33)  8(26) | 9(29)  20(67)  22(71) |
| **12. I know who I might see at the hospital and why.**  V1  V2  V3 | 29  28  29 | 0  0  0 | 1(3)  0  1(3) | 6(21)  7(25)  7(23) | 22(76)  21(75)  21(70) | 31  30  31 | 0  0  0 | 1(3)  1(3)  0 | 11(35)  4(13)  6(19) | 19(61)  25(83)  25(81) |
| **13. I know how to pronounce bronchiectasis.**  V1  V2  V3 | 28  28  29 | 0  0  0 | 3(11)  1(4)  1(3) | 4(14)  4(14)  5(17) | 21(75)  23(82)  23(79) | 31  30  31 | 0  0  0 | 0  0  0 | 7(23)  4(13)  4(13) | 24(77)  26(87)  27(87) |
| **14. I can explain about my bronchiectasis to others.**  V1  V2  V3 | 29  28  29 | 1(3)  2(7)  0 | 3(10)  3(11)  5(17) | 11(38)  17(61)  10(34) | 14(48)  6(21)  14(48) | 31  30  31 | 1(3)  0  0 | 5(16)  3(10)  1(3) | 12(39)  8(27)  10(32) | 13(42)  19(63)  20(65) |
| **15. I feel I can cope with and live with my bronchiectasis.**  V1  V2  V3 | 29  28  29 | 0  0  0 | 2(7)  5(18)  2(7) | 16(55)  17(61)  18(62) | 11(38)  6(21)  9(30) | 31  30  31 | 0  0  0 | 2(6)  1(3)  2(6) | 19(61)  12(40)  12(39) | 10(32)  17(57)  17(55) |

Table 7. Bronchiectasis Knowledge Questionnaire multiple response questions.

|  | **n** | **Control group n and (%)** | | |  | **Intervention group n and (%)** | | |
| --- | --- | --- | --- | --- | --- | --- | --- | --- |
| **Question/Visit** |  | **Answered correctly** | **Answered incorrectly** | **Didn’t know** |  | **Answered correctly** | **Answered Incorrectly** | **Didn’t know** |
| **1. Bronchiectasis is always caused by smoking. (F)**  V1  V2  V3 | 29  28  29 | 22(76)  27(96)  24(83) | 0  0  1(3) | 7(24)  1(4)  4(14) | 31  30  30 | 28(90)  29(97)  30(100) | 0  0  0 | 3(10)  1(3)  0 |
| **2. Bronchiectasis is the same as chronic obstructive pulmonary disease (COPD) (F)**  V1  V2  V3 | 29  28  29 | 17(59)  18(64)  20(69) | 2(7)  3(11)  2(7) | 9(31)  7(25)  7(24) | 31  30  30 | 18(58)  23(77)  24(80) | 4(13)  0  2(7) | 9(29)  7(23)  4(13) |
| **3. Bronchiectasis cannot be cured in most cases but can be managed or controlled. (T)**  V1  V2  V3 | 29  28  29 | 28(97)  28(100)  25(86) | 0  0  2(7) | 1(3)  0  2(7) | 31  30  30 | 30(97)  30(100)  30(100) | 1(3)  0  0 | 0  0  0 |
| **4. Bronchiectasis can be cured in most cases. (F)**  V1  V2  V3 | 29  28  29 | 25(86)  27(96)  24(83) | 0  0  1(3) | 4(14)  1(4)  4(14) | 31  30  30 | 29(94)  29(97)  30(100) | 0  0  0 | 2(6)  1(3)  0 |
| **5. Fatigue or extreme tiredness can be a symptom of bronchiectasis. (T)**  V1  V2  V3 | 29  28  29 | 20(69)  23(82)  24(83) | 0  0  1(3) | 9(31)  5(17)  4(14) | 31  30  30 | 23(74)  30(100)  29(97) | 1(3)  0  1(3) | 7(23)  0  0 |
| **6. Antibiotics for bronchiectasis chest infections should be taken for 5 days and then stopped. (F)**  V1  V2  V3 | 29  28  29 | 25(86)  24(86)  25(86) | 1(3)  1(4)  2(7) | 3(10)  3(11)  2(7) | 31  30  30 | 29(94)  24(80)  27(90) | 0  2(7)  2(7) | 2(6)  4(13)  1(3) |
| **7. Antibiotics are *always* needed if I am coughing phlegm. (F)**  V1  V2  V3 | 29  28  29 | 20(69)  20(71)  22(76) | 6(21)  5(18)  4(14) | 3(10)  3(11)  3(10) | 31  30  30 | 26(84)  23(77)  23(77) | 4(13)  2(7)  2(7) | 1(3)  5(17)  5(17) |
| **8. Chest clearance, breathing exercises or physio are only required during an infection. (F)**  V1  V2  V3 | 29  28  29 | 27(93)  25(89)  25(86) | 0  1(4)  2(7) | 2(7)  2(7)  2(7) | 31  30  30 | 29(94)  29(97)  29(97) | 0  1(3)  1(3) | 2(6)  0  0 |
| **9. It is recommended that I should have a flu jab every year. (T)**  V1  V2  V3 | 29  28  29 | 28(97)  28(100)  28(97) | 0  0  0 | 1(3)  0  1(3) | 31  30  30 | 31(100)  30(100)  30(100) | 0  0  0 | 0  0  0 |
| **10. I should not exercise if I have bronchiectasis. (F)**  V1  V2  V3 | 29  28  29 | 24(83)  23(82)  27(93) | 0  2(7)  2(7) | 5(17)  3(11)  0 | 31  30  30 | 27(87)  29(97)  29(97) | 1(3)  0  0 | 3(10)  1(3)  1(3) |
| **11. Patients with bronchiectasis sometimes cough up blood when they have a chest infection. (T)**  V1  V2  V3 | 29  28  29 | 14(48)  14(50)  16(55) | 5(17)  4(14)  4(14) | 10(34)  10(36)  9(31) | 31  30  30 | 18(58)  30(100)  25(83) | 2(6)  0  0 | 11(35)  0  5(17) |

Table 8. Bronchiectasis Knowledge Questionnaire True, False questions.
